# Extracellular Vesicles as Dynamic Sensors of Redox–Inflammatory Balance: Potential Implications for Aging in Healthy Subjects

**DOI:** 10.3390/biomedicines14061317

**Published:** 2026-06-10

**Authors:** Irene Martínez de Toda, Rafael Moreno-Gómez-Toledano, Julia Carracedo, Mónica De la Fuente, Rafael Ramírez-Carracedo

**Affiliations:** 1 Department of Genetics, Physiology and Microbiology (Unit of Animal Physiology), Faculty of Biological Sciences, Complutense University of Madrid (UCM), 28040 Madrid, Spain; 2 Institute of Investigation Hospital 12 Octubre (imas12), 28041 Madrid, Spain; 3 RICORS2040-Renal, Instituto de Salud Carlos III (ISCIII), 28029 Madrid, Spain; 4 University of Alcalá, Area of Human Anatomy and Embryology, Department of Surgery, Medical and Social Sciences, School of Medicine and Health Sciences, 28871 Alcalá de Henares, Spain; 5 Centro de Investigación Biomédica en Red de Enfermedades Cardiovasculares (CIBERCV), Instituto de Salud Carlos III (ISCIII), 28029 Madrid, Spain

**Keywords:** extracellular vesicles, aging, immunosenescence, oxidative stress

## Abstract

**Background/Objectives:** Chronological age does not fully capture the heterogeneity of physiological aging among healthy individuals. Immune aging and redox imbalance are key hallmarks of biological aging, yet their interaction and relationship with circulating extracellular vesicles (EVs) remain incompletely understood. This study aimed to investigate whether endothelial- and platelet-derived EVs are associated with immune and oxidative aging processes in clinically healthy subjects. **Methods:** Circulating EVs were isolated and characterized by flow cytometry in a cohort of healthy volunteers spanning a wide age range. Endothelial-derived EVs (EeEVs) and platelet-derived EVs (PEVs) were quantified and analyzed in relation to chronological age, immune function parameters, redox biomarkers, ImmunolAge (an immune aging index), and OxyScore (a composite redox index). A normalized EV-Score was developed using an age- and sex-adjusted Z-score approach. Associations were assessed using correlation analyses, non-linear regression models, generalized additive models, and receiver operating characteristic (ROC) curves. **Results:** Both EeEVs and PEVs increased non-linearly with age, with a pronounced rise during midlife. EV concentrations were positively associated with molecular aging markers and inversely related to multiple immune function parameters. EVs were also linked to redox biomarkers, although oxidative status alone did not explain EV variability. EV-Score was strongly associated with immune aging and showed context-dependent relationships with oxidative status. Notably, high EV-Score values were observed primarily in individuals with accelerated immune aging, whereas subjects with high oxidative status but preserved immune aging exhibited low EV-Score values. ROC analyses demonstrated that the discriminative capacity of EV-Score for immune or oxidative aging depended on the combined immune–redox context. **Conclusions:** Circulating EVs may reflect the integrated state of immune and redox aging rather than chronological age alone. These findings suggest the potential utility of EVs as dynamic biomarkers of biological aging in healthy individuals and highlight the importance of considering immune and oxidative processes jointly to interpret EV-associated aging signatures.

## 1. Introduction

According to the United Nations World Population Prospects 2024 report, population aging represents an inevitable global demographic shift, particularly evident in developing regions [1]. The proportion of individuals aged 65 years and older continues to increase due to sustained improvements in life expectancy combined with declining birth rates. This demographic transition poses major biomedical and socioeconomic challenges and highlights the need to identify molecular and cellular mechanisms underlying healthy aging and distinguish it from pathological aging.

Aging has traditionally been associated with an increased prevalence of chronic non-communicable diseases [2], and marked interindividual variability exists even among clinically healthy older adults. Lifestyle and intrinsic factors can modulate aging-associated dysfunctions, supporting the concept of healthy aging, which emphasizes the maintenance of physiological integrity and adaptive capacity at the cellular and molecular levels [2,3].

Chronic low-grade inflammation and redox imbalance are widely recognized as central hallmarks of aging. Immunosenescence is characterized by impaired innate and adaptive immune responses, increased susceptibility to infections, reduced vaccine efficacy, and a higher incidence of cancer and autoimmune diseases [4]. In parallel, aging is accompanied by a persistent pro-inflammatory state known as inflammaging, which contributes to tissue damage and functional decline. These immune alterations have been proposed as indicators of biological aging, often providing more informative insights than chronological age alone [2,5].

In this context, the Immunity Clock has emerged as a quantitative approach to estimate biological age through immune function assessment. This model integrates parameters related to immune cell responsiveness and functionality to calculate ImmunolAge, a numerical estimate of biological aging that correlates with oxidative stress, frailty, morbidity, and survival [6,7]. Oxidative stress, in turn, plays a key role in aging by promoting mitochondrial dysfunction, cellular senescence, and immune dysregulation, establishing the oxi-inflamm-aging cycle [2,5,8].

Circulating Extracellular Vesicles (EVs) have recently gained attention not only as minimally invasive biomarkers but also as potential functional mediators of aging-related processes. EVs are actively secreted by cells under specific physiological and pathological conditions and participate in intercellular communication through the transfer of bioactive cargo, including proteins, lipids, and nucleic acids. Consequently, their cellular origin, molecular cargo, and surface protein composition vary according to tissue type, pathological state, and aging status [9]. Depending on their cargo and surface proteins, EVs have been shown to modulate several signaling pathways and biological processes, including activation of inflammatory pathways such as NF-κB [10], as well as fibrosis- and aging-associated signaling mechanisms [11].

It has been demonstrated that EVs reflect the functional state of their cells of origin. In particular, endothelial-derived EVs (EEVs) and platelet-derived EVs (PEVs) are closely associated with vascular homeostasis, inflammation, and redox balance, and both their circulating concentrations and molecular cargo undergo age-related alterations [12,13,14,15]. Understanding the relationship between immune function, oxidative status, and EV subpopulations across the human lifespan may provide mechanistic insights into aging processes and facilitate the identification of novel circulating biomarkers with potential clinical relevance.

While most EV-related aging studies focus on pathological conditions, their behavior in healthy individuals and during normal biological aging remains understudied. In this cross-sectional study, we analyzed circulating EeEVs and PEVs in clinically healthy individuals aged 20 to 102 years, together with their immunological and oxidative status as central pillars of aging, to explore their association with biological aging.

## 2. Materials and Methods

### 2.1. Human Cohort and Age Group Classification

This cross-sectional observational study included a total of 141 volunteers (92 women and 49 men), aged between 20 and 102 years. Participants were stratified into five experimental groups according to World Health Organization (WHO) age classifications: Young Age (20–45 years; 20 women and 13 men), Middle Age (45–60 years; 25 women and 13 men), Elderly Age (60–75 years; 11 women and 11 men), Senile Age (75–90 years; 16 women and 4 men), and Long Livers (≥90 years; 20 women and 8 men).

All participants were clinically healthy at the time of enrollment, with no evidence of acute or chronic disease, as determined by medical history, physical examination, and routine laboratory analyses. Exclusion criteria included obesity; active smoking or excessive alcohol consumption; the presence of metabolic, cardiovascular, respiratory, or inflammatory disorders (including diabetes mellitus, asthma, or cardiovascular disease); organ dysfunction; and the use of medications or dietary supplements known to influence oxidative stress, inflammatory status, or extracellular vesicle-related parameters.

Immune function and redox-related parameters included in the present study were obtained within the same recruitment framework and during the same general study period, following identical inclusion and exclusion criteria for healthy volunteers. Due to the progressive incorporation of different experimental approaches and the availability of biological samples, not all participants underwent every determination. Consequently, some individuals had complete datasets, whereas others only had immune function- or redox-related measurements available. Importantly, participant inclusion in each analysis was determined by sample availability and technical feasibility rather than by biological or clinical characteristics, reducing the likelihood of systematic selection bias. A supplementary flow diagram detailing participant distribution and sample sizes across the different analyses performed in the study is provided in Supplementary S1.

All volunteers provided written informed consent authorizing the use of biological samples for research purposes. The study was conducted in accordance with the principles of the Declaration of Helsinki and was approved by the local Scientific Ethics Committee.

### 2.2. Human Sample Preparation

Peripheral blood samples were collected by antecubital venipuncture into citrate-containing tubes. Whole blood and plasma fractions were obtained by centrifugation at 1300× *g* for 20 min. Plasma and whole blood aliquots were stored at −80 °C until analysis.

### 2.3. Extracellular Vesicle Isolation and Characterization

Prior to characterization, EVs were isolated from platelet-free plasma by a two-step centrifugation protocol. A first centrifugation at 2000× *g* for 5 min was performed to pellet and discard cell debris and large vesicles. Subsequently, samples were centrifuged at 11,000× *g* for 35 min to obtain a pellet enriched in EVs.

EV characterization was performed by flow cytometry according to the International Society for Extracellular Vesicles (ISEV2023) guidelines (MISEV2023) [16] as described previously [17]. Optimal fluorescence settings for EV analysis were established using Sphero™ Nanofluorescent Particle Size Standard calibration beads (0.22, 0.45, 0.88, and 1.32 µm) from Spherotech (Lake Forest, IL, USA). Appropriate unstained and isotype controls were used to establish gating thresholds and define positive events. Flow cytometry analyses and gating strategies were performed as previously validated and described in detail by Valera et al. [17]. Channel overlap shown in figures was performed post-acquisition for visualization only, whereas gating was performed on the original fluorescence channels.

Although complementary biophysical characterization methods such as nanoparticle tracking analysis (NTA) or transmission electron microscopy (TEM) were not performed in the present study, the analytical strategy was based on previously validated protocols for the identification of circulating endothelial- and platelet-derived EV populations. In addition, given that CD31 is not exclusively expressed by endothelial cells, CD31^+^CD41^−^ EVs were interpreted as endothelial-enriched EVs (EeEVs) rather than as definitively endothelial-derived vesicles.

### 2.4. ImmunolAge Assessment

Biological age associated with immune system function (ImmunolAge) was calculated using the algorithm previously described by Martínez de Toda et al. [6]. This composite index integrates multiple immune function parameters known to change with aging and to reflect immunosenescence.

Peripheral blood samples were used to assess immune function parameters, including Natural Killer (NK) activity, phytohemagglutinin (PHA)-stimulated lymphoproliferation, neutrophil chemotaxis, neutrophil phagocytic index and lymphocyte chemotaxis, following standardized procedures as previously reported [6]. All measurements were performed under identical experimental conditions to minimize inter-assay variability.

The ImmunolAge value was calculated by applying the published formula, which weights individual immune parameters to generate a single numerical estimate of immune-related biological age. Higher ImmunolAge values indicate a more advanced immune aging profile relative to chronological age.

### 2.5. OxyScore Determination

Systemic oxidative status was evaluated in whole blood samples using the OxyScore, a composite index previously described by Veglia et al. [18]. This score integrates pro-oxidant and antioxidant biomarkers to provide a global estimate of redox balance.

Individual oxidative stress markers were first standardized using Z-score transformation. Antioxidant markers were used to calculate a protection score (PS), defined as the mean of standardized antioxidant parameters, while pro-oxidant markers were used to calculate a damage score (DS), defined as the mean of standardized pro-oxidant parameters. The OxyScore was subsequently calculated by subtracting the protection score from the damage score (OxyScore = DS − PS), with higher values indicating a more pro-oxidative systemic state.

When biomarker distributions showed high dispersion, logarithmic transformation was applied prior to standardization. Antioxidant markers included catalase (CAT), glutathione reductase (GR), glutathione peroxidase (GPx), and reduced glutathione (GSH). Pro-oxidant markers included malondialdehyde (MDA), xanthine oxidase (XO), and the oxidized-to-reduced glutathione ratio (GSSG/GSH). Catalase, xanthine oxidase, malondialdehyde and the components of the glutathione cycle were measured as described previously [17,19,20].

### 2.6. EV-Score Determination

For each subject, EeEV and PEV concentrations were independently standardized using Z-score normalization within sex- and age-specific strata according to the formula: Z = (X − μ)/σ, where X represents the individual EV concentration, μ the mean value of the corresponding subgroup, and σ the standard deviation. The EV-Score was subsequently calculated as the arithmetic mean of standardized EeEV and PEV values.

### 2.7. Protein Carbamylation Assessment

Protein carbamylation was measured in plasma and quantified using the OxiSelect™ Protein Carbamylation Sandwich ELISA Kit (Cell Biolabs, San Diego, CA, USA), following the manufacturer’s instructions. Carbamylated protein levels were expressed as concentration (ng/mL) and subsequently normalized to total protein content to obtain a carbamylation ratio (carbamylated proteins/total protein). This normalization was applied to minimize the influence of potential differences in total protein concentration among samples.

### 2.8. Statistical Analysis

Statistical analyses were conducted in accordance with the STROBE recommendations for cross-sectional studies. Data distribution was assessed for each dataset using the Shapiro–Wilk normality test. Based on the results, appropriate parametric or nonparametric statistical methods were applied.

The dataset was compiled from previously conducted studies with differing data availability; consequently, not all variables were measured in all participants. Variations in sample size between analyses are therefore attributable to missing data for parameters required to calculate the combined scores. Individuals lacking one or more required parameters were excluded only from the relevant analyses. No selective or arbitrary inclusion criteria were applied, and all analyses were performed using available data for each variable.

For group comparisons, Kruskal–Wallis tests or two-way analysis of variance (ANOVA) were performed using GraphPad Prism software 8 (GraphPad Software, San Diego, CA, USA). When significant main effects or interactions were detected, post hoc multiple-comparison analyses were conducted using Tukey’s test. Pairwise comparisons were performed using Student’s *t*-test or the Mann–Whitney test, as appropriate. Pearson correlation analyses between quantitative variables were conducted using two-tailed tests.

Nonlinear regression analyses were performed using a four-parameter logistic (4PL) model in GraphPad Prism. To explore complex relationships between variables, generalized additive models (GAMs) with bidimensional spline functions were applied using the R 4.5 statistical environment (R Foundation for Statistical Computing, Vienna, Austria).

Receiver operating characteristic (ROC) curve analyses were performed using GraphPad Prism to evaluate the diagnostic performance of extracellular vesicles as biomarkers. A *p*-value < 0.05 was considered statistically significant.

Detailed statistical information associated with graphical analyses was additionally compiled in Supplementary S2 to improve figure readability.

### 2.9. Use of Artificial Intelligence Tools

Generative Artificial Intelligence (ChatGPT 5.5) tools were used in a limited manner to assist with the technical implementation of statistical analyses and language editing. Specifically, an AI-based language model was consulted to support the coding and structuring of generalized additive models (GAMs) with bidimensional splines in the R environment, and to improve the clarity and readability of the English language in the manuscript. The choice of statistical approach, model specification, parameter selection, data analysis, and biological interpretation of the results were entirely performed by the authors. All code was reviewed, adapted, and executed by the research team, and no AI tools were used to generate data, figures, or scientific conclusions. The authors reviewed and approved the final manuscript and remain fully responsible for its content.

## 3. Results

### 3.1. Circulating EeEVs and PEVs Increase with Age and Show Sex-Specific Dynamics

**Figure 1:**
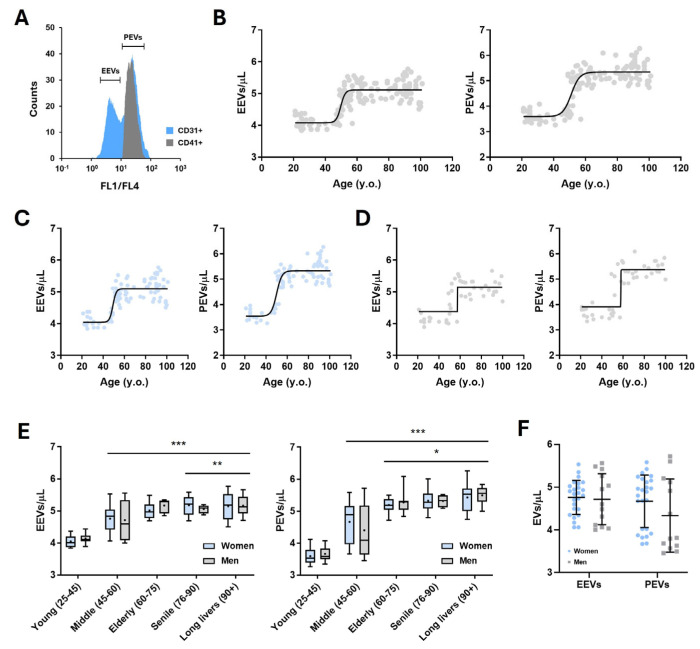
Circulating EVs dynamics in healthy human volunteers. (**A**) Representative flow cytometry analysis of circulating extracellular vesicles (EVs), showing the identification of endothelial-derived EVs (EeEVs; CD31^+^CD41^−^) and platelet-derived EVs (PEVs; CD31^+^CD41^+^). Fluorescence channels FL1 and FL4 were overlapped during post-analysis exclusively for visualization purposes, to facilitate the simultaneous identification of EV subpopulations; quantitative analyses were performed on the original, non-overlapped channels. (**B**) Total circulating EV concentration plotted against chronological age in the overall population. (**C**,**E**) EV concentration as a function of age in women. (**D**,**E**) EV concentration as a function of age in men. (**F**) Distribution of circulating EV concentrations in the Middle Age group (45–60 years old). Each dot represents an individual subject. Statistical significance is * *p* < 0.05 vs. Middle, ** *p* < 0.01 vs. Middle, *** *p* < 0.001 vs. Young. Total *n* = 141 (92 women, 49 men); Middle Age *n* = 38 (25 women and 13 men).

Circulating EVs from 141 healthy volunteers were isolated and characterized by flow cytometry to distinguish endothelial-enriched derived EVs (EeEVs; CD31^+^CD41^−^) from platelet-derived EVs (PEVs; CD31^+^CD41^+^) (Figure 1A). This strategy allowed the reliable identification of major EV subpopulations present in peripheral blood.

Total circulating EV concentration was first quantified and analyzed in relation to chronological age. When all participants were considered together, EV levels increased significantly with advancing age, with a marked rise observed from middle age (45–60 years old) onwards (Figure 1B). This age-associated increase was evident in both women and men when analyzed separately (Figure 1C–E). To model the relationship between EV concentration and age, a four-parameter logistic (4PL) regression was applied, as EV levels exhibited a clear non-linear, sigmoidal increase across the lifespan. In combined data and in women-only analyses, EV accumulation followed a gradual trajectory characterized by a moderate hill slope. In contrast, men displayed a markedly steeper slope, indicating a more abrupt increase in EV concentration during midlife.

This sex-specific pattern was further supported by the greater dispersion of EV values observed among middle-aged men compared with women of the same age range (Figure 1F), which may indicate greater inter-individual variability and a sharper transition in circulating EV levels in males during the 40–60-year period.

### 3.2. Circulating EeEVs and PEVs Are Associated with Markers of Molecular Aging, Immune Function, and Redox Balance

Circulating endothelial-derived (EeEV) and platelet-derived EV (PEV) concentrations were individually correlated with a panel of biomarkers related to molecular aging, immune function, and redox homeostasis (Table 1). To validate the association between EVs and aging, EV concentrations were compared with circulating levels of carbamylated proteins, a recognized marker of molecular aging. Both EeEVs and PEVs showed a significant positive correlation with carbamylated proteins, as indicated by Pearson correlation coefficients.

Several parameters of immune function were inversely associated with circulating EV levels. In particular, NK cell activity, PHA-stimulated lymphocyte proliferation, and neutrophil and lymphocyte chemotaxis were negatively correlated with both EeEVs and PEVs. These associations suggest that higher circulating EV concentrations may be linked to reduced immune functional capacity.

EV concentrations were also significantly associated with multiple redox-related biomarkers. Positive correlations were observed between EV levels and markers of oxidative damage, including malondialdehyde (MDA) and oxidized glutathione (GSSG). In parallel, EVs were positively correlated with several antioxidant enzymes and molecules, such as catalase (CAT), glutathione reductase (GR), glutathione peroxidase (GPx), and reduced glutathione (GSH). Conversely, a significant negative association was observed between EV concentrations and xanthine oxidase (XO) activity. While contradictory, upregulation of antioxidant systems often accompanies increased oxidative burden, especially in healthy aging.

Collectively, these findings suggest that circulating EVs may be closely associated with molecular aging, alterations in immune function, and redox imbalance, which is consistent with their involvement in aging-related biological processes.

### 3.3. ImmunolAge Reveals Immune-Related Biological Aging and Explains Midlife Changes in Circulating EVs

Immune system function reflects underlying molecular mechanisms associated with physiological aging that are not always captured by chronological age. Therefore, validated immune-based indices such as the Immunity Clock, quantified here as the ImmunolAge parameter, provide an alternative measure of biological aging.

**Figure 2:**
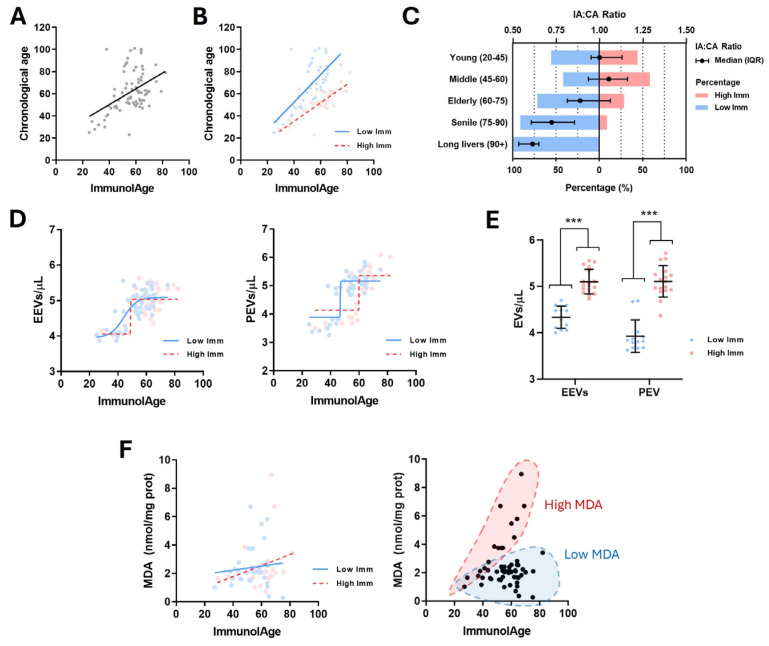
ImmunolAge determination and association with circulating EVs. (**A**) Correlation between ImmunolAge and chronological age in the overall population (*r* = 0.4498; *p* < 0.0001). (**B**) ImmunolAge plotted against chronological age after stratification into subjects with a decelerated aging rate (Low Imm; *r* = 0.6817; *p* < 0.0001) and an accelerated aging rate (High Imm; *r* = 0.8703; *p* < 0.0001). (**C**) Distribution of IA:CA ratios across age groups. (**D**) Circulating endothelial-derived EVs and platelet-derived EVs plotted against ImmunolAge in DAR and AAR subpopulations. Curves represent four-parameter logistic (4PL) regression fits. (**E**) EeEV and PEV concentrations in chronologically middle-aged subjects (45–60 years), stratified by immunological aging status (DAR vs. AAR). (**F**) Relationship between ImmunolAge and malondialdehyde (MDA) levels, showing the absence of a significant linear correlation but the presence of distinct high- and low-MDA subpopulations. Each dot represents an individual subject. Statistical significance is indicated as *** *p* < 0.001. Total *n* = 80 (46 Low Imm, 34 High Imm); Middle age *n* = 30 (13 Low Imm, 17 High Imm); MDA-ImmunolAge *n =* 59 (36 Low Imm, 23 High Imm).

Using the immune function parameters described above, ImmunolAge was calculated for 80 subjects and compared with chronological age. A significant positive correlation was observed between both parameters (*r* = 0.4498; *p* < 0.0001), suggesting that ImmunolAge increases with advancing chronological age (Figure 2A). To further refine this relationship at the individual level, the ratio between ImmunolAge and chronological age (IA:CA) was calculated. Subjects were classified as presenting a Decelerated Aging Rate (DAR; IA:CA < 1) or an Accelerated Aging Rate (AAR; IA:CA > 1).

As shown in Figure 2B, both DAR and AAR subpopulations exhibited significant positive correlations between ImmunolAge and chronological age. However, DAR subjects displayed a steeper slope with a moderate correlation (*r* = 0.6817; *p* < 0.0001), whereas AAR subjects showed a more diagonal slope and a stronger association (*r* = 0.8703; *p* < 0.0001), meaning that individuals with decelerated aging maintain a comparatively lower biological age, particularly at older chronological ages. Consistently, the lowest IA:CA ratios were predominantly observed in subjects aged ≥65 years, whereas higher ratios were more frequently detected in the middle-aged group (Figure 2C and Supplementary S3).

Circulating EV concentrations were subsequently analyzed within DAR and AAR subpopulations using ImmunolAge as the independent variable (Figure 2D). Four-parameter logistic (4PL) regression models were applied due to the non-linear relationship observed. EV concentrations increased with rising ImmunolAge, particularly within the 40–60 ImmunolAge range. Notably, endothelial-derived EVs (EeEVs) displayed distinct patterns between subgroups: DAR subjects showed a gradual increase, whereas AAR subjects exhibited a sharp rise in EeEV levels. In contrast, platelet-derived EVs (PEVs) showed comparable trajectories in both DAR and AAR groups, suggesting that EeEVs are more sensitive to immune aging status, whereas PEVs may reflect a generalized ImmunolAge-associated increase.

Given the pronounced increase in EV concentrations observed in chronologically middle-aged individuals, EV levels were further analyzed within the 45–60-year age group after stratification by immunological aging status (Figure 2E). Both EeEVs and PEVs were significantly higher in AAR subjects compared with DAR subjects, indicating that elevated ImmunolAge is associated with the midlife surge in circulating EVs.

Finally, to explore whether ImmunolAge was associated with other aging-related biological processes, linear regression analyses were performed between ImmunolAge and redox-related biomarkers. No significant linear correlation was observed between ImmunolAge and malondialdehyde (MDA) levels in either DAR or AAR groups (Figure 2F). However, data dispersion revealed distinct subpopulations characterized by high or low MDA levels, suggesting that oxidative status and immune aging may be partially uncoupled. These observations prompted a more detailed analysis of oxidative stress parameters, as described in the following section.

### 3.4. Systemic Oxidative Status Is Associated with Immune Aging and Extracellular Vesicle Levels

Oxidative stress is a well-recognized hallmark of aging, reflecting cellular senescence and the progressive decline of organ function, and it is known to be interconnected with other physiological processes, including immune function. The association observed between ImmunolAge and MDA in Figure 2F suggested that oxidative status and immune aging may be related in a subset of individuals. Therefore, systemic oxidative status was assessed using the OxyScore to explore this relationship in greater depth.

**Figure 3:**
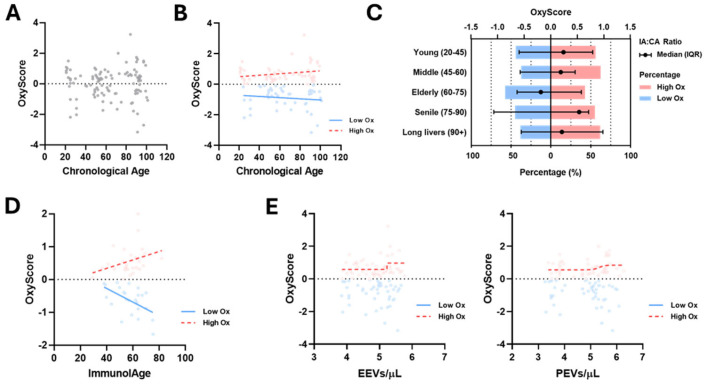
OxyScore determination and association with circulating EVs. (**A**) Association between systemic oxidative status, expressed as OxyScore, and chronological age in the overall population. (**B**) Linear regression analysis of OxyScore stratified by oxidative status, showing subjects with low OxyScore (Low Ox) and high OxyScore (High Ox). (**C**) Distribution of subjects with low and high OxyScore values across age groups. (**D**) Correlation between ImmunolAge and OxyScore stratified by oxidative status, revealing opposite trends in low (*r* = −0.534; *p* = 0.007) and high (*r* = 0.375; *p* = 0.044) OxyScore subgroups. (**E**) Four-parameter logistic (4PL) regression analysis of circulating endothelial-derived extracellular vesicles (EeEVs) and platelet-derived extracellular vesicles (PEVs) in subjects with low and high OxyScore values. Total *n* = 103 (46 Low Ox, 57 High Ox); OxyScore-ImmunolAge *n =* 53 (24 Low Ox, 29 High Ox).

OxyScore values were first analyzed in relation to chronological age. In contrast to ImmunolAge, no significant correlation was detected between OxyScore and chronological age (Figure 3A). Nevertheless, individuals were stratified into two subgroups based on their OxyScore values: subjects with low oxidative status (OxyScore < 0) and those with high oxidative status (OxyScore > 0). Although opposite trends were observed, showing a slight positive tendency in the high OxyScore group and a slight negative tendency in the low OxyScore group, these associations did not reach statistical significance (Figure 3B).

The distribution of subjects with high and low OxyScore values across age groups is shown in Figure 3C. Except for the elderly group (60–75 years old), all age groups displayed median OxyScore values above zero. As OxyScore is calculated using a Z-score-based methodology adjusted for age and sex, this pattern is expected and reflects relative positioning within the population rather than the presence of pathological oxidative imbalance, particularly considering that all participants were clinically healthy. Notably, an approximately equal distribution of individuals with low and high oxidative status was observed within each age group.

Given the potential link between immune function and oxidative stress, the relationship between OxyScore and ImmunolAge was subsequently examined. A significant association was detected when subjects were stratified by oxidative status (Figure 3D). Individuals with high OxyScore values showed a positive correlation between OxyScore and ImmunolAge (*r* = 0.3754; *p* = 0.0447), whereas those with low OxyScore values exhibited a significant negative correlation (*r* = −0.534; *p* = 0.0072). These opposing trends suggest that impaired immune function may correlate with a worse oxidative profile, even among clinically healthy individuals.

Finally, to evaluate whether circulating EVs reflect systemic oxidative status, four-parameter logistic (4PL) regression analyses were performed between EV concentrations and OxyScore (Figure 3E). No significant association was observed in the low OxyScore subgroup. In contrast, subjects with high OxyScore values showed a marked increase in circulating EVs, with endothelial-derived EeEVs displaying a steeper slope (4.177) than PEVs (3.878). These results indicate that circulating EVs, particularly EeEVs, may serve as sensitive biomarkers of elevated oxidative stress in healthy individuals.

### 3.5. Integration of Extracellular Vesicles with Immune and Redox Aging Through a Composite EV-Score

As shown in the previous results, EeEVs and PEVs display highly similar age-related behaviors and may be related to both immune aging and oxidative status in clinically healthy individuals. Based on these observations, we sought to integrate EV levels into a single normalized parameter in order to evaluate their potential as composite biomarkers of biological aging.

To this end, an EV-Score was developed to normalize circulating EV levels (EeEVs and PEVs combined) relative to age- and sex-matched reference values. The EV-Score is based on a modified Z-score approach, calculated as:

$$EV-Score=\frac{{\bar{X}}_{EEVs,PEVs}-\mu_{age,sex}}{\sigma_{age,sex}}$$

To ensure appropriate normalization, subjects were first stratified by sex and age group prior to EV-Score calculation, accounting for the known influence of these variables on EV release.

**Figure 4:**
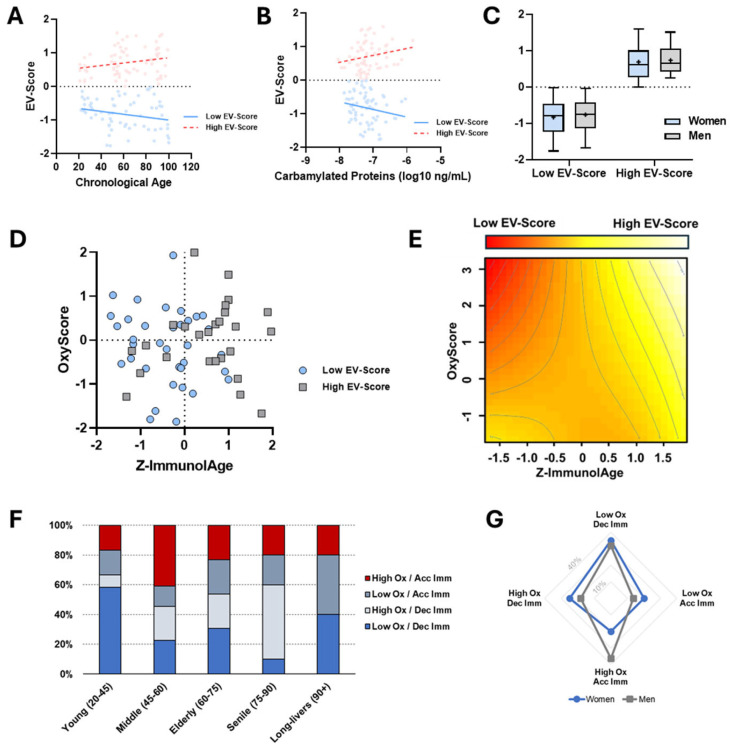
EV-Score calculation and association with OxyScore and ImmunolAge. (**A**) Linear regression between EV-Score and chronological age. Subjects were stratified into Low EV-Score (*r* = −0.2338; *p* = 0.0294) and High EV-Score (*r* = 0.2137; *p* = 0.0401) groups, showing opposite age-associated trends. (**B**) Association between EV-Score and circulating carbamylated protein levels, a marker of molecular aging, stratified by EV-Score subgroup (High: *r* = 0.2258; *p* = 0.0352; Low: *r* = −0.2236; *p* = 0.0356). (**C**) Comparison of EV-Score distributions between women and men in Low and High EV-Score groups, showing no significant sex differences after normalization. (**D**) Three-dimensional relationship between standardized ImmunolAge (Z-ImmunolAge) and OxyScore, highlighting individual EV-Score classification (Low EV-Score, blue dots; High EV-Score, grey squares). (**E**) Generalized additive model (GAM) with bidimensional splines representing the continuous EV-Score surface across the OxyScore–Z-ImmunolAge space, illustrating regions of low and high EV-Score values (*p* = 0.000194). (**F**) Distribution of subjects across OxyScore–ImmunolAge quadrants stratified by chronological age groups. Quadrants are defined as Low/High OxyScore (Ox) and Accelerated/Decelerated ImmunolAge (Imm). (**G**) Sex-specific distribution of subjects across OxyScore–ImmunolAge quadrants (women, blue circles; men, grey squares). Each symbol represents an individual subject. Total *n* = 134 (66 Low EV-Score, 68 High EV-Score); Quadrant and GAM analysis *n* = 62 (40 women, 22 men).

Figure 4A shows the linear regression between EV-Score and chronological age. As in previous analyses, subjects were categorized according to whether their EV-Score was positive (High EV-Score) or negative (Low EV-Score). A significant positive association was observed between chronological age and EV-Score in the High EV-Score subgroup (*r* = 0.2137; *p* = 0.0401), whereas an inverse association was detected in individuals with Low EV-Scores (*r* = −0.2338; *p* = 0.0294), indicating divergent EV trajectories with aging that are consistent with previous results.

To further validate EV-Score as a marker of biological aging, its association with circulating carbamylated proteins, a recognized indicator of molecular aging, was examined (Figure 4B). Consistent with previous findings obtained for EeEVs and PEVs individually, subjects with High EV-Scores exhibited a positive correlation with carbamylated protein levels (*r* = 0.2258; *p* = 0.0352), while Low EV-Score individuals showed a significant negative association (*r* = −0.2236; *p* = 0.0356). These results support EV-Score as a potential integrative marker of molecular aging.

Given the known influence of sex on aging trajectories, EV-Scores were compared between men and women prior to further analyses (Figure 4C). No significant differences were detected between sexes in either the Low or High EV-Score groups, supporting the use of EV-Score as a sex-independent parameter after normalization.

Previous results demonstrated an interaction between immune aging and oxidative status in this cohort. To explore whether EV-Score reflects this interaction, a three-variable analysis was performed integrating EV-Score, ImmunolAge, and OxyScore. Figure 4D depicts the distribution of individuals according to OxyScore and standardized ImmunolAge values (Z-ImmunolAge), indicating whether subjects were part of the Low EV-Score (blue dots) or the High EV-Score (grey squares) groups (Figure 4D). To facilitate interpretation of these complex relationships, and confirm statistical significance of the results, a generalized additive model (GAM) with bidimensional splines was applied, yielding a continuous EV-Score surface (color-coded) across the OxyScore–ImmunolAge space (Figure 4E).

Both representations show that elevated EV-Scores are predominantly observed in individuals with Z-ImmunolAge values above zero, indicating that higher EV levels may be associated with accelerated immune aging, in agreement with the previous results. Notably, individuals displaying a high oxidative status but low ImmunolAge values consistently exhibited low EV-Scores. This pattern is evident in the upper-left quadrant of Figure 4D, where nearly all subjects belong to the Low EV-Score subgroup. Collectively, these findings suggest that EV levels may increase primarily in association with immune aging, whereas elevated oxidative stress alone, when immune aging is decelerated, is not sufficient to drive high EV-Scores. Accordingly, subjects with low oxidative burden and low ImmunolAge present higher EV levels than those with high oxidative status but preserved immune aging. These observations support the concept that EVs may act as dynamic sensors whose circulating levels reflect the combined state of immune and redox aging rather than either process in isolation.

To further contextualize these observations, the distribution of individuals across the four OxyScore–ImmunolAge quadrants was analyzed according to chronological age and sex. Quadrants were defined as follows: High or Low OxyScore combined with Accelerated or Decelerated ImmunolAge. Figure 4F shows the age group distribution. Interestingly, both young adults (20–45 years) and long-lived individuals (≥90 years) displayed the highest proportions of subjects with low oxidative status and decelerated ImmunolAge (≈40–60%). The senile group (75–90 years) exhibited the greatest proportion of individuals with high oxidative status but decelerated immune aging. In contrast, the middle-aged group (45–60 years) showed the highest percentage of subjects with concurrent high oxidative stress and accelerated ImmunolAge, indicating a convergence of adverse aging processes in this age window.

Finally, sex-specific distributions across these quadrants are shown in Figure 4G. Women were more frequently represented in the Low OxyScore/Decelerated ImmunolAge group (35%), whereas men most commonly clustered in the High OxyScore/Accelerated ImmunolAge quadrant (36.36%). Nonetheless, a substantial proportion of men also fell within the Low OxyScore/Decelerated ImmunolAge group (31.81%), underscoring the heterogeneity of aging trajectories within each sex.

### 3.6. Context-Dependent Discriminative Capacity of EV-Score for Immune and Oxidative Aging

Finally, to further explore the potential of EVs as dynamic sensors of immune and redox aging, receiver operating characteristic (ROC) curve analyses were performed to evaluate the ability of EV-Score to discriminate ImmunolAge and OxyScore levels.

**Figure 5:**
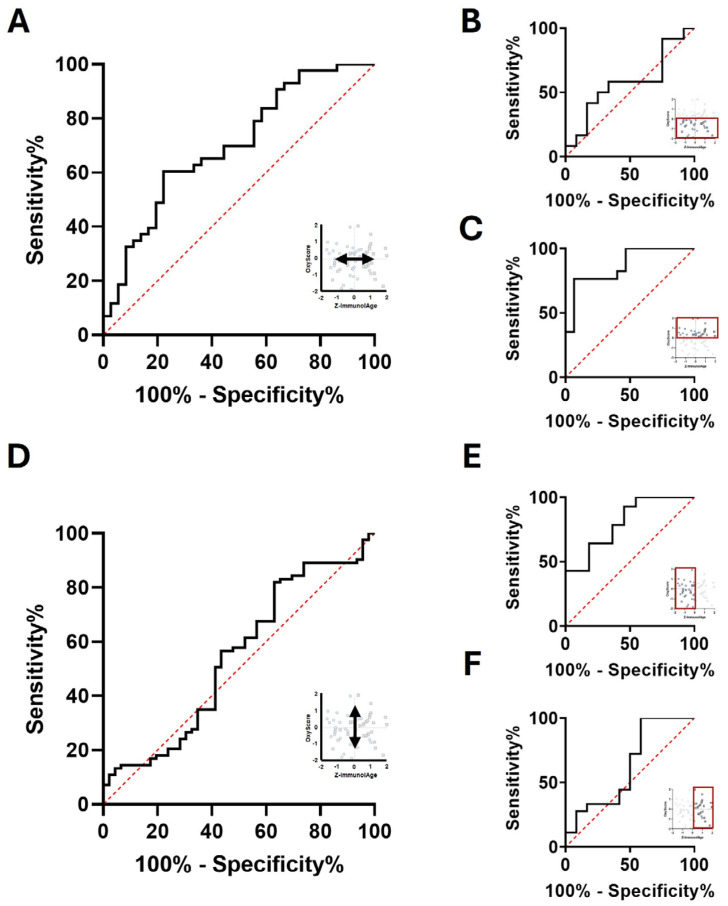
ROC Curve analysis. (**A**) ROC curve showing the capacity of EV-Score to discriminate low versus high ImmunolAge in the whole cohort (AUC = 0.7003; *p* = 0.0023). (**B**,**C**) ROC curves assessing EV-Score discrimination of ImmunolAge in subjects with (**B**) low (**C**) or high oxidative status (AUC = 0.8667; *p* = 0.0004). (**D**) ROC curve evaluating the ability of EV-Score to discriminate low versus high OxyScore in the whole cohort. (**E**,**F**) ROC curves assessing (**E**) EV-Score discrimination of OxyScore in individuals with decelerated immune aging (AUC = 0.8052; *p* = 0.0101) or (**F**) accelerated immune aging. ImmunolAge prediction *n* = 79; OxyScore prediction *n* = 132.

First, the discriminative capacity of EV-Score for immune aging was evaluated in the whole cohort by comparing individuals EV levels with low or high ImmunolAge. In agreement with previous analyses, EV-Score showed a significant ability to distinguish immune aging status (AUC = 0.7003; *p* = 0.0023; Figure 5A), which may suggest a close association between EV levels and immune aging.

As earlier results suggested that oxidative status modulates EV behavior, the same ROC analyses were then stratified according to OxyScore. In individuals with low oxidative status (OxyScore < 0), EV-Score showed limited capacity to discriminate ImmunolAge (Figure 5B). In contrast, in subjects with high oxidative status (OxyScore > 0), EV-Score displayed a markedly improved discriminative performance (AUC = 0.8667; *p* = 0.0004; Figure 5C), suggesting that the association between EVs and immune aging becomes stronger under conditions of increased oxidative burden.

The reciprocal analysis was subsequently performed to assess the capacity of EV-Score to discriminate oxidative status. When all subjects were analyzed together, EV-Score showed poor discrimination between low and high OxyScore groups (Figure 5D), in line with the weaker direct associations observed previously between EVs and oxidative markers.

However, when individuals were stratified by immune aging status, a different pattern emerged. In subjects with decelerated immune aging (Z-ImmunolAge < 0), EV-Score showed a significant ability to discriminate oxidative status (AUC = 0.8052; *p* = 0.0101; Figure 5E). Conversely, EV-Score showed limited discriminative power in individuals with accelerated immune aging (Figure 5F).

Together, these results reinforce the concept that EV behavior may be highly context-dependent, with their discriminative capacity varying according to the combined immune and oxidative aging profile of the individual. Rather than reflecting a single biological process, EVs appear to integrate multiple aging-related signals, supporting their role as potential dynamic biomarkers of physiological aging in healthy subjects.

## 4. Discussion

Aging is a complex and multifactorial process driven by interconnected molecular, cellular, and physiological alterations that progressively impair function and increase disease susceptibility [21]. Because individuals age at different rates, biological age has emerged as a more informative descriptor than chronological age, reflecting the cumulative burden of molecular damage and systemic dysregulation [2,5]. Biological age can be estimated through several approaches, including DNA methylation-based epigenetic clocks [22,23], immune-derived indices such as ImmunolAge [6,7], and composite biomarker panels integrating clinical and molecular parameters [24].

Extracellular vesicles (EVs) are key mediators of intercellular communication, transporting proteins, lipids, and nucleic acids that reflect the physiological state of their parental cells [25]. Increasing evidence indicates that EVs participate in processes closely associated with aging, including oxidative stress, immunosenescence, and inflammaging [15,26].

Altered EV secretion and cargo composition have been described in senescent cells and aged tissues, suggesting that EVs may represent both hallmarks of aging and potential therapeutic targets [15,26,27]. Recent evidence also indicates that EV-associated cargo can actively modulate senescence-related pathways involved in endothelial dysfunction [28], macrophage activation through NF-κB signaling [10], and fibrosis- and aging-associated processes mediated by the TGF-β1/Smad3 pathway [11]. Conversely, certain aging-associated EV populations may exert compensatory immunoregulatory effects and attenuate hyperinflammatory states in older individuals [29]. Among them, endothelial-derived EVs (EeEVs) increase during aging and chronic inflammation and contribute to vascular dysfunction [30], while platelet-derived EVs (PEVs) accumulate in aged plasma and may promote thrombosis and endothelial injury [31]. However, their behavior across the lifespan in healthy individuals remains poorly characterized.

In the present study, EeEV and PEV concentrations were quantified in plasma from 141 clinically healthy individuals aged between 20–102 years, including long-lived participants without diagnosed disease. This design enabled EV dynamics to be examined across the lifespan in the absence of overt pathology, covering the entire aging process beginning in the third decade of life.

Consistent with previous reports [30,32,33], in this study circulating EV levels increased significantly with chronological age. However, most prior studies have relied on categorical comparisons (e.g., young vs. old), which may overlook subtler dynamics across the lifespan. In contrast, the present work employed a continuous, non-categorical approach to assess the association between circulating EV levels and chronological age. Continuous analysis revealed a non-linear trajectory characterized by accelerated accumulation during midlife (approximately 40–60 years) followed by a stabilization thereafter. Although average EV concentrations did not differ significantly between sexes, greater variability was observed among men aged 40–60 years. This dispersion may reflect sex-specific physiological influences, including declining androgen levels and the partial vascular protection provided by estrogens before menopause [34]. Experimental evidence further indicates that sex hormones, sex chromosomes, and sex-dependent inflammatory set-points can influence EV biogenesis and release [35,36], although direct evidence in EeEVs and PEVs remains limited.

To determine whether EV levels reflect biological aging rather than chronological age alone, immune and oxidative parameters were analyzed using the ImmunolAge and OxyScore indices. ImmunolAge reflects immune system functionality and has been associated with lifespan and oxidative status [6,7]. After calculation, subjects were classified as presenting accelerated aging (AAR; IA:CA > 1) or decelerated aging (DAR; IA:CA < 1), and interestingly most AAR individuals were located between 20 and 75 years, with the highest prevalence in the 45–60 age range. This distribution may reflect subclinical physiological alterations that precede overt disease. Indeed, longitudinal studies indicate that changes in immune function, low-grade inflammation, and molecular damage often emerge during midlife and can remain clinically silent for years [37]. As previously stated, this study included only clinically healthy individuals, and the presence of diagnosed diseases was an exclusion criterion. Consequently, individuals in the oldest groups likely represent strictly healthy subjects, whereas some younger or middle-aged participants may still harbor undetected conditions. Although these would not affect their classification as clinically healthy, they could nonetheless influence analytical parameters.

Within this framework, EV concentrations increased significantly with ImmunolAge. Interestingly, this increase was steeper in AAR subjects for both analyzed EV populations, whereas individuals with a DAR showed a flatter increase in EeEVs. This pattern suggests that EeEV release may be more sensitive to subtle immunological alterations, while PEV release may be pronounced in both conditions. This different behavior could be partly explained because platelet EV releasing has been shown to be stimulated not only by inflammation but also by platelet senescence associated with chronological aging [38], while endothelial EV liberation is much more dependent on the vascular environment and pathological stimuli [39].

Prior results showed an unexplained elevation and strong dispersion of EV levels in individuals aged 40–65 years. To investigate this, subjects in the middle-age group were reanalyzed according to their immune profile. Unlike the analysis based solely on chronological age, significant differences in EeEV and PEV release were detected between AAR and DAR subgroups. These findings may indicate that the elevation previously observed in chronologically classified middle-aged individuals was largely driven by immunological status. Stratification based on ImmunolAge, adjusted for chronological age, clarified the variability and revealed distinct patterns between groups, highlighting that immune aging may contribute substantially to EV dynamics during this critical midlife window.

Building on these observations, the relationship between immune aging and oxidative stress was examined to determine whether EV variability might also reflect redox balance. Plasma malondialdehyde (MDA) levels showed no overall correlation with immune status, although two subpopulations were apparent: individuals in whom oxidative and immune alterations occurred independently, and others in whom elevated ImmunolAge coincided with increased oxidative stress. This suggests that, while immune aging seems to be a key driver of EV release, its interplay with redox balance is heterogeneous across individuals. Such divergence has been described previously and may give rise to distinct immune–oxidative aging phenotypes [40], motivating a more systematic assessment of oxidative status.

To better characterize oxidative balance, a composite OxyScore integrating pro-oxidant and antioxidant markers was calculated and normalized for age and sex. Only a weak association between OxyScore and chronological age was observed. Interestingly, elderly individuals frequently displayed antioxidant-dominant profiles, whereas younger and older groups tended to show mildly pro-oxidant conditions. This pattern may reflect physiological redox signaling rather than pathological oxidative stress, as only a pronounced imbalance would indicate potential pathological alterations [41]. When analyzed in relation to ImmunolAge, oxidative status showed a clearer association. Some individuals maintained low oxidative stress despite increasing ImmunolAge, whereas others exhibited elevated oxidative stress alongside immune aging, highlighting that immune and redox aging, although mechanistically linked, do not necessarily progress at the same rate.

EV levels showed a weaker association with oxidative status than with immune aging. Nevertheless, higher EV concentrations were observed in individuals with elevated oxidative stress, consistent with evidence that reactive oxygen species stimulate EV secretion [42]. Mitochondrial ROS have also been shown to enhance EV release under metabolic or hypoxic stress [43], suggesting that oxidative imbalance may amplify EV production in cells already exposed to inflammatory or metabolic challenges.

To integrate these findings, a composite EV-Score was generated by combining normalized EeEV and PEV values. This score reflected the EV patterns observed in this cohort, including the strong association with chronological age and the presence of subpopulations with either high or low EV levels. These groups also showed similar patterns in circulating carbamylated proteins, a marker of molecular aging associated with oxidative damage and age-related pathology [20].

Combining EV-Score, OxyScore, and normalized ImmunolAge revealed four physiological profiles within the cohort. Individuals with high EV-Scores were predominantly located in the high ImmunolAge group and frequently exhibited elevated oxidative stress, suggesting a potential link between oxidative imbalance, immune aging, and EV release. When oxidative status remained balanced, however, EV concentrations were less informative as markers of immune aging. These observations are consistent with the view that EVs may function as dynamic sensors of systemic physiological states, integrating signals from inflammation and oxidative stress. However, it is important to remark that the observed stratification should be understood in the context of a relatively small cohort of clinically healthy individuals and may not directly extend to broader epidemiological settings.

Age-stratified analyses further showed that unfavorable oxidative and immunological profiles were most prevalent in middle-aged individuals (45–60 years), whereas long-lived participants displayed patterns similar to the youngest group. This observation supports the concept that midlife represents a critical transition period in systemic physiological regulation [37]. The most deleterious profiles were also more frequent in men, consistent with known sex differences in immune and inflammatory aging [5,44,45,46,47].

These results indicate that EeEVs and PEVs may be actively and dynamically released in healthy individuals in response to underlying metabolic and mechanistic processes, potentially reflecting subclinical alterations. The EV-Score captures this behavior, with ROC analyses showing it predicts ImmunolAge more effectively than oxidative status when considered independently. Notably, predictive performance depends on the interplay between immune and oxidative conditions: EVs are elevated in individuals with both high OxyScore and high ImmunolAge but remain low in those with high OxyScore and preserved immune function. However, given the subgroup sample sizes and exploratory nature of the analyses, the reported ROC-derived thresholds should be interpreted cautiously until validated in independent cohorts.

This pattern likely reflects the integration of cellular stress responses and immune competence. In individuals with concurrent oxidative stress and immune aging, multiple pro-inflammatory and stress pathways are active, promoting endothelial and platelet activation and enhancing EV biogenesis [48]. Conversely, robust immune function may limit EV release under oxidative challenge through compensatory mechanisms such as efficient antioxidant defenses, repair pathways, or controlled apoptosis [49]. These findings highlight the context-dependent nature of EV release, which may reflect not only the level of stress but also the organism’s capacity to respond and maintain homeostasis.

Several limitations should be noted. First, complementary biophysical EV characterization techniques such as nanoparticle tracking analysis (NTA) or transmission electron microscopy (TEM) were not performed. Therefore, the present findings should be interpreted within the context of flow cytometry-based immunophenotypic analysis of circulating EV subpopulations. In addition, EV analysis focused on vesicle concentration and cellular phenotype without evaluating vesicular cargo or release mechanisms. Future studies incorporating orthogonal EV characterization approaches together with proteomic, lipidomic, or RNA profiling will be important to further define the biological significance of EV dynamics during aging. Residual confounding cannot be excluded. Variables including BMI, menopausal status, dietary habits, physical activity, platelet count, lipid profile, and subclinical vascular alterations may influence EV release and were not uniformly available for adjustment in the present cohort.

Furthermore, EV identification was based on a CD31/CD41 marker strategy, in which CD31^+^CD41^−^ EVs were operationally considered enriched in endothelial origin. However, because CD31 is not exclusively expressed by endothelial cells, this population cannot be interpreted as purely endothelial-derived vesicles. Thus, incorporation of additional endothelial-specific markers such as CD144, CD146, or CD105 in future studies would improve EV subpopulation specificity.

In addition, as this was an exploratory cross-sectional study, the EV-Score was internally derived from the study cohort and was not externally validated. Independent and longitudinal validation studies will therefore be necessary to determine its robustness and potential applicability as a biomarker of biological aging.

Finally, the sample size, particularly among long-lived participants, was limited, and not all participants underwent every analysis due to sample availability and the progressive incorporation of experimental determinations during the study period. Moreover, the cross-sectional design precludes evaluation of longitudinal changes or causal relationships.

## 5. Conclusions

Endothelial- and platelet-derived EVs emerge as potential dynamic indicators of systemic physiological status, integrating signals from immune function, oxidative balance, and other age-related processes. Unlike static markers of chronological age, the studied EV populations appear to be associated with the combined effects of immune aging and oxidative stress, potentially capturing transient or tissue-specific perturbations that conventional molecular clocks may miss. The EV-Score suggests that these vesicles may reflect subclinical alterations, highlighting their potential utility as exploratory integrative biomarkers of biological aging.

The main intention of this study is not to propose a single biomarker of aging but to show that EV profiling may provide complementary insight into interconnected physiological processes, offering additional perspective on individual aging trajectories and age-related physiological alterations.

However, given the exploratory nature of the present study and the absence of external validation, these findings should be confirmed in independent and longitudinal cohorts. Nevertheless, the present results support the growing concept that circulating EVs may provide a unique integrative perspective on the complex interplay between immune function, redox homeostasis, and healthy aging.

## Figures and Tables

**Table 1:** Associations between circulating extracellular vesicles and biomarkers of molecular aging, immune function, and redox status.

Variable	EeEVs/μL		PEVs/μL		*n*
	Pearson *r*	*p* (Two-Tailed)	Pearson *r*	*p* (Two-Tailed)	
Carbamylated Proteins (ng/mL)	0.19	0.023	0.20	0.021	139
NK (%lysis)	−0.43	0.016	−0.48	0.0067	30
PHA stimulated lymphocytes (cpm)	−0.21	0.042	−0.20	0.049	94
Neutrophil Chemotaxis (cells/mm^2^)	−0.44	0.016	−0.45	0.013	30
Lymphocyte Chemotaxis (cells/mm^2^)	−0.55	0.0017	−0.47	0.009	30
CAT (U/mg prot)	0.30	0.0059	0.36	0.0008	83
Gr (U/mg prot)	0.37	0.0002	0.42	<0.0001	94
GPx (U/mg prot)	0.36	0.0006	0.39	0.0002	86
GSH (nmol/mg prot)	0.29	0.027	0.34	0.0085	60
GSSG (nmol/mg prot)	0.32	0.012	0.35	0.0062	60
MDA (nmol/mg prot)	0.22	0.027	0.21	0.03	106
XO (mU/mg prot)	−0.35	0.0073	−0.32	0.013	59

## Data Availability

The raw data supporting the conclusions of this article will be made available by the authors on request.

## Supplementary Materials

The following supporting information can be downloaded at: https://www.mdpi.com/article/10.3390/biomedicines14061317/s1, Supplementary S1: Flowchart of study population selection and sample size distribution (*n*) across study stages; Supplementary S2: Additional statistical information; Supplementary S3: Immunolage subpopulation determination.

## Abbreviations

The following abbreviations are used in this manuscript:

EVs	Extracellular Vesicles
EeEVs	Endothelial-enriched Extracellular Vesicles
PEVs	Platelet Extracellular Vesicles
NK	Natural Killer
PHA	Phytohemagglutinin
AAR	Accelerated Aging Rate
DAR	Decelerated Aging Rate
HOS	High Oxidative Status
LOS	Low Oxidative Status
