# Supplementary material for: Extracellular Vesicles as Dynamic Sensors of Redox–Inflammatory Balance: Potential Implications for Aging in Healthy Subjects
Source: Biomedicines. 2026 Jun 10;14(6):1317. doi: 10.3390/biomedicines14061317 (PMC13297170; doi:10.3390/biomedicines14061317)

Supplementary 1. Flowchart of study population selection and sample size distribution (n) across study stages.

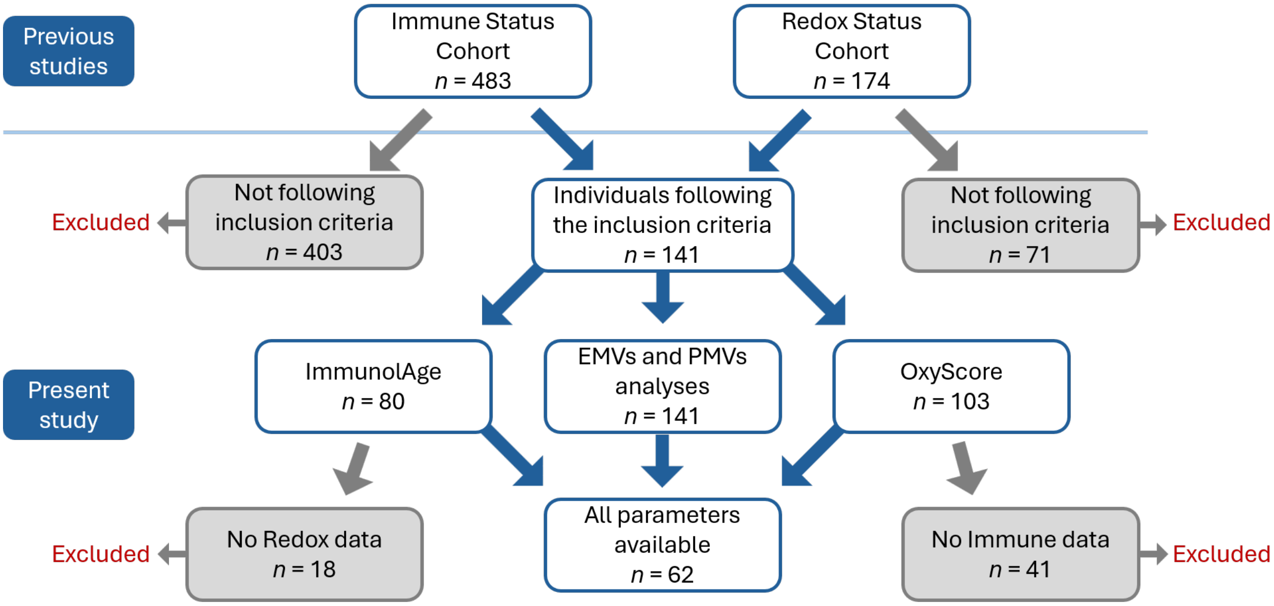

Supplementary 2. Additional statistical information.

Figure 1B

	EEVs	PEVs
Top (A)	5.11	5.343
Bottom (D)	4.079	3.589
LogIC50 (C)	49.74	51.03
HillSlope (B)	0.3133	0.1634
R^2	0.6798	0.7622

Figure 1C

Women	EEVs	PEVs
Top (A)	5.1	3.542
Bottom (D)	4.042	5.331
LogIC50 (C)	49.18	49.99
HillSlope (B)	0.2783	0.175
R^2	0.6778	0.7886

Figure 1D

Men	EEVs	PEVs
Top (A)	5.145	5.374
Bottom (D)	4.377	3.912
LogIC50 (C)	57.46	57.95
HillSlope (B)	42.57	6.388
R^2	0.5673	0.7042

Figure 2D

EEVs	Low Imm	High Imm
Top (A)	3.953	5.035
Bottom (D)	5.09	4,060
LogIC50 (C)	44.06	48,93
HillSlope (B)	0.09462	5,267
R^2	0.5288	0.5021

PEVs	Low Imm	High Imm
Top (A)	5.163	5.356
Bottom (D)	3.892	4.135
LogIC50 (C)	46,84	59,95
HillSlope (B)	4,504	6,824
R^2	0.691	0.5842

Figure 3E

	EEVs	PMVs
Top (A)	0.9691	0.844
Bottom (D)	0.5855	0.5558
LogIC50 (C)	~5,228	5.323
HillSlope (B)	4.177	3.878
R^2	0.1218	0.06871

Figure 5

Figure	AUC	p	95% CI	Cutoff	Sensitivity (%)	Specificity (%)
5A	0.7003	0.0023	0,5846 to 0,8159	> 0.08500	60.47	77.78
5B	0.5764	0.5254	0,3382 to 0,8146	> 0.4875	50	75
5C	0.8667	0.0004	0,7407 to 0,9926	> 0.07350	76.47	93.33
5D	0.5419	0.4314	0,4345 to 0,6493	> -0.6080	81.93	36.96
5E	0.8052	0.0101	0,6333 to 0,9771	< -0.05900	92.86	54.55
5F	0.6296	0.2359	0,4092 to 0,8500	> -0.6945	100	41.67

Supplementary 3: Immunolage subpopulation determination

Immunolage subpopulation determination

Aging Rate = IA/CA

Rate<1: Decelerated Aging Rate

Rate >1: Accelerated Aging Rate

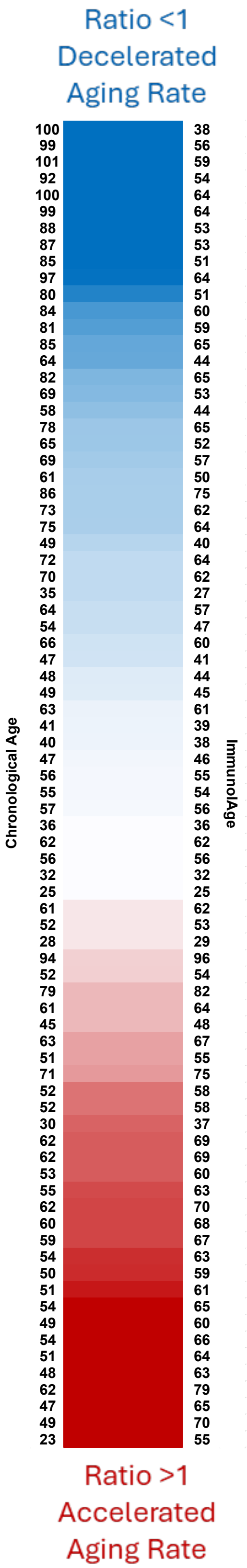

Supplement: Supplementary file 1 [file biomedicines-14-01317-s001.zip › biomedicines-4310117-supplementary.pdf]
